# 5′′-(4-Chloro­benzyl­idene)-4′-(4-chloro­phen­yl)-5-fluoro-1′,1′′-dimethyl­indoline-3-spiro-2′-pyrrolidine-3′-spiro-3′′-piperidine-2,4′′-dione

**DOI:** 10.1107/S1600536811007550

**Published:** 2011-03-05

**Authors:** J. Kalyana Sundar, B. Devi Bala, S. Natarajan, J. Suresh, P. L. Nilantha Lakshman

**Affiliations:** a Department of Physics, Madurai Kamaraj University, Madurai 625 021, India; b Department of Organic Chemistry, Madurai Kamaraj University, Madurai 625 021, India; c Department of Physics, The Madura College, Madurai 625 011, India; d Department of Food Science and Technology, University of Ruhuna, Mapalana, Kamburupitiya 81100, Sri Lanka

## Abstract

The piperidine ring of the title compound, C_30_H_26_Cl_2_FN_3_O_2_, adopts a twisted chair conformation. The pyrrolidine ring has a twisted envelope structure with the N atom at the flap [displaced by 0.592 (3) Å]. The fluoro­oxindole, chloro­phenyl and chloro­benzyl­idene groups are planar with r.m.s. deviations of 0.0348, 0.0048 and 0.0048 Å, respectively. The structure is stabilized by inter­molecular N—H⋯O hydrogen bonds.

## Related literature

For biological applications of 1,4-dihydro­pyridine derivatives, see: Jerom & Spencer (1988 ▶); Perumal *et al.* (2001 ▶); Hagenbach & Gysin (1952 ▶); Mobio *et al.* (1989 ▶); Katritzky & Fan (1990 ▶); Ganellin & Spickett (1965 ▶); El-Subbagh *et al.* (2000 ▶). For their use as synthetic inter­mediates in the preparation of various pharmaceuticals, see: Wang & Wuorola (1992 ▶). For their ocurrence in natural products such as alkaloids, see: Angle & Breitenbucher (1995 ▶).
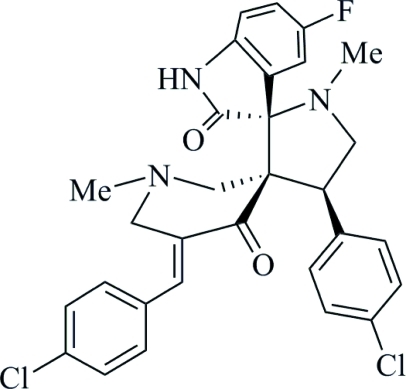


## Experimental

### 

#### Crystal data

C_30_H_26_Cl_2_FN_3_O_2_
*M* *_r_* = 550.44
Monoclinic, 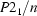

*a* = 16.694 (3) Å
*b* = 8.705 (4) Å
*c* = 18.474 (3) Å
β = 103.27 (4)°
*V* = 2613.3 (14) Å^3^
*Z* = 4
Mo *K*α radiation
μ = 0.29 mm^−1^
*T* = 293 K
0.23 × 0.21 × 0.18 mm

#### Data collection

Nonius MACH3 diffractometer
Absorption correction: ψ scan (North *et al.*, 1968 ▶) *T*_min_ = 0.936, *T*_max_ = 0.950
5427 measured reflections
4581 independent reflections
2891 reflections with *I* > 2σ(*I*)
*R*_int_ = 0.020
3 standard reflections every 60 min  intensity decay: none

#### Refinement

*R*[*F* ^2^ > 2σ(*F* ^2^)] = 0.036
*wR*(*F* ^2^) = 0.104
*S* = 1.02
4581 reflections
349 parameters
H atoms treated by a mixture of independent and constrained refinement
Δρ_max_ = 0.29 e Å^−3^
Δρ_min_ = −0.33 e Å^−3^

### 

Data collection: *CAD-4 EXPRESS* (Enraf–Nonius, 1994 ▶); cell refinement: *CAD-4 EXPRESS*; data reduction: *XCAD4* (Harms & Wocadlo, 1996 ▶); program(s) used to solve structure: *SHELXS97* (Sheldrick, 2008 ▶); program(s) used to refine structure: *SHELXL97* (Sheldrick, 2008 ▶); molecular graphics: *PLATON* (Spek, 2009 ▶); software used to prepare material for publication: *SHELXL97*.

## Supplementary Material

Crystal structure: contains datablocks global, I. DOI: 10.1107/S1600536811007550/zj2003sup1.cif

Structure factors: contains datablocks I. DOI: 10.1107/S1600536811007550/zj2003Isup2.hkl

Additional supplementary materials:  crystallographic information; 3D view; checkCIF report

## Figures and Tables

**Table 1:** Hydrogen-bond geometry (Å, °)

*D*—H⋯*A*	*D*—H	H⋯*A*	*D*⋯*A*	*D*—H⋯*A*
N3—H1*N*⋯O1^i^	0.84 (3)	2.50 (3)	3.288 (3)	157 (3)

Symmetry code: (i) 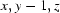.

**Fig. 1.**
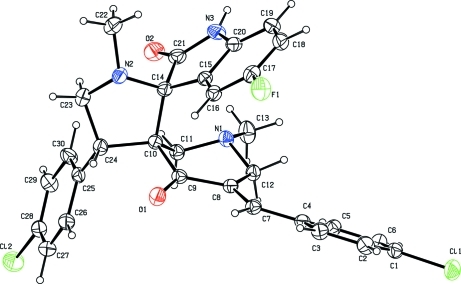
The molecular structure of (I), showing 30% probability displacement ellipsoids and the atom-numbering scheme.

**Fig. 2.**
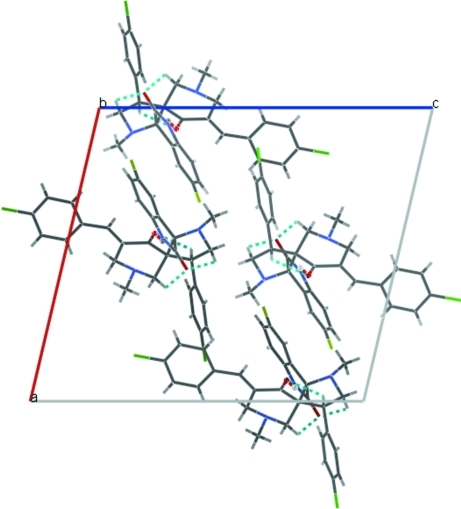
Packing diagram

## supplementary crystallographic information

### Comment

In the family of heterocyclic compounds, nitrogen containing heterocycles especially substituted piperidin-4-ones have considerable importance due to their variety of biological properties such as analgesic (Jerom *et al.*, 1988), local anaesthetic (Perumal *et al.*, 2001; Hagenbach & Gysin, 1952), antimicrobial, bactericidal, fungicidal, herbicidal, anticancer, *CNS* stimulant and depressant activities (Mobio *et al.*, 1989; Katritzky & Fan, 1990; Ganellin & Spickett, 1965) and antiviral, antitumour (El-Subbagh *et al.*, 2000). Also they are important synthetic intermediates in the preparation of various pharmaceuticals (Wang & Wuorola, 1992) and widely prevalent in natural products such as alkaloids (Angle & Breitenbucher, 1995). Hence, the present X-ray crystallographic study of the title compound has been carried out to determine the conformation of the system.

The piperidine ring of the title compound, C_30_H_26_N_3_O_2_Cl_2_F, adopts a twisted chair conformation (C8/C9/C10/C11/N1/C12). Pyrrolidine ring has the twisted envelope structure with N atom at the flap (0.592 (3)Å from the mean plane formed by the atoms C10/C14/C23/C24) and this orientation may be influenced by the intramolecular C23—H23A···O2 hydrogen bond (Table 1).

Fluorooxindole, the chlorophenyl and chlorophenylmethylidine groups are planar as confirmed by thevalues of the r.m.s. deviation (0.0348 Å, 0.0048Å and 0.0048 Å), respectively,from the mean planes of the above groups. Flurooxindole is inclined with the plane of chlorophenyl by 33.99 (2)° and 55.56 (2)° with the mean plane of chlorophenylmethilidine. The sum of the bond angles around N1 atom (334.22°) of the piperidine ring in the molecule is in accordance with the *sp*^2^ hybridization. Further, the structure is stabilized by intermolecular N—H···O hydrogen bond and intramolecular C—H···O hydrogen bonds.

### Experimental

A mixture of 1-methyl-3,5-bis[(*E*)-chlorobenzylidene]tetrahydro-4 (1*H*)-pyridin-ones (1 mmol), 5-fluoroisatin (1 mmol) and sarcosine in methanol (10 ml) was refluxed for 30 min. After completion of the reaction as evident from TLC, the mixture was poured into water (50 ml). The precipitated solid was filtered and washed with water to obtain the pure product. The product was dissolved in methonol and allowed to evoporate at room temperature. Transparent, needle-shaped, colourless crystals of small sizes (8 *x* 2 *x* 2 mm^3^)were obtained in a period of about a week. Yield:94%; *M*.p:224 °C

### Refinement

H atoms were placed at calculated positions and allowed to ride on their carrier atoms with C—H = 0.93–0.97 Å, and *U*_iso_ = 1.2*U*_eq_(C) for CH_2_ and CH groups and *U*_iso_ = 1.5*U*_eq_(C) for CH_3_ group.The N-bound H atom is located in a difference Fourier map and its positional parameters were refined.

### Crystal data

**Table:**

C_30_H_26_Cl_2_FN_3_O_2_	*F*(000) = 1144
*M**_r_* = 550.44	*D*_x_ = 1.399 Mg m^−^^3^
Monoclinic, *P*2_1_/*n*	Mo *K*α radiation, λ = 0.71073 Å
Hall symbol: -P 2yn	Cell parameters from 25 reflections
*a* = 16.694 (3) Å	θ = 2–25°
*b* = 8.705 (4) Å	µ = 0.29 mm^−^^1^
*c* = 18.474 (3) Å	*T* = 293 K
β = 103.27 (4)°	Block, colourless
*V* = 2613.3 (14) Å^3^	0.23 × 0.21 × 0.18 mm
*Z* = 4	

### Data collection

**Table:**

Nonius MACH3 diffractometer	2891 reflections with *I* > 2σ(*I*)
Radiation source: fine-focus sealed tube	*R*_int_ = 0.020
graphite	θ_max_ = 25.0°, θ_min_ = 2.3°
ω–2θ scans	*h* = 0→19
Absorption correction: ψ scan (North *et al.*, 1968)	*k* = −1→10
*T*_min_ = 0.936, *T*_max_ = 0.950	*l* = −21→21
5427 measured reflections	3 standard reflections every 60 min
4581 independent reflections	intensity decay: none

### Refinement

**Table:**

Refinement on *F*^2^	Primary atom site location: structure-invariant direct methods
Least-squares matrix: full	Secondary atom site location: difference Fourier map
*R*[*F*^2^ > 2σ(*F*^2^)] = 0.036	Hydrogen site location: inferred from neighbouring sites
*wR*(*F*^2^) = 0.104	H atoms treated by a mixture of independent and constrained refinement
*S* = 1.02	*w* = 1/[σ^2^(*F*_o_^2^) + (0.0445*P*)^2^ + 0.726*P*] where *P* = (*F*_o_^2^ + 2*F*_c_^2^)/3
4581 reflections	(Δ/σ)_max_ = 0.001
349 parameters	Δρ_max_ = 0.29 e Å^−^^3^
0 restraints	Δρ_min_ = −0.33 e Å^−^^3^

### Special details

**Table:**

Geometry. All e.s.d.'s (except the e.s.d. in the dihedral angle between two l.s. planes) are estimated using the full covariance matrix. The cell e.s.d.'s are taken into account individually in the estimation of e.s.d.'s in distances, angles and torsion angles; correlations between e.s.d.'s in cell parameters are only used when they are defined by crystal symmetry. An approximate (isotropic) treatment of cell e.s.d.'s is used for estimating e.s.d.'s involving l.s. planes.
Refinement. Refinement of *F*^2^ against ALL reflections. The weighted *R*-factor *wR* and goodness of fit *S* are based on *F*^2^, conventional *R*-factors *R* are based on *F*, with *F* set to zero for negative *F*^2^. The threshold expression of *F*^2^ > σ(*F*^2^) is used only for calculating *R*-factors(gt) *etc*. and is not relevant to the choice of reflections for refinement. *R*-factors based on *F*^2^ are statistically about twice as large as those based on *F*, and *R*- factors based on ALL data will be even larger.

### Fractional atomic coordinates and isotropic or equivalent isotropic displacement parameters (Å^2^)

**Table:**

	*x*	*y*	*z*	*U*_iso_*/*U*_eq_	
Cl1	0.15668 (5)	0.37573 (8)	0.72167 (3)	0.0831 (2)	
Cl2	−0.36431 (5)	0.61752 (10)	0.00265 (4)	0.0919 (3)	
N1	−0.05322 (10)	0.13961 (19)	0.27962 (9)	0.0464 (4)	
O1	0.07132 (10)	0.52336 (18)	0.24553 (8)	0.0613 (4)	
C8	0.03804 (12)	0.3538 (2)	0.33420 (11)	0.0419 (5)	
F1	0.32228 (9)	0.21475 (19)	0.36806 (8)	0.0808 (5)	
N3	0.05530 (14)	−0.1040 (2)	0.21711 (12)	0.0603 (6)	
C12	−0.02137 (13)	0.2314 (2)	0.34544 (11)	0.0473 (5)	
H12A	0.0060	0.1641	0.3854	0.057*	
H12B	−0.0671	0.2797	0.3608	0.057*	
C9	0.03991 (12)	0.4022 (2)	0.25700 (11)	0.0435 (5)	
O2	−0.05184 (11)	−0.0289 (2)	0.12232 (10)	0.0724 (5)	
N2	0.08528 (11)	0.2056 (2)	0.11641 (9)	0.0524 (5)	
C20	0.12500 (14)	−0.0377 (3)	0.26298 (13)	0.0511 (6)	
C15	0.12839 (13)	0.1176 (2)	0.24568 (11)	0.0448 (5)	
C5	0.04926 (13)	0.3405 (3)	0.50697 (12)	0.0521 (6)	
H5	0.0001	0.3012	0.4789	0.063*	
C10	−0.00213 (12)	0.2963 (2)	0.19252 (11)	0.0420 (5)	
C11	−0.07990 (13)	0.2364 (2)	0.21440 (11)	0.0459 (5)	
H11A	−0.1125	0.3216	0.2256	0.055*	
H11B	−0.1132	0.1776	0.1739	0.055*	
C4	0.10401 (13)	0.4086 (2)	0.47049 (11)	0.0451 (5)	
C17	0.25566 (14)	0.1306 (3)	0.33291 (13)	0.0570 (6)	
C25	−0.10489 (14)	0.4406 (2)	0.08703 (11)	0.0475 (5)	
C28	−0.26410 (16)	0.5504 (3)	0.03646 (13)	0.0592 (6)	
C24	−0.01808 (13)	0.3837 (3)	0.11635 (11)	0.0487 (5)	
H24	0.0175	0.4746	0.1241	0.058*	
C14	0.05642 (13)	0.1582 (2)	0.18243 (11)	0.0464 (5)	
C16	0.19562 (13)	0.2034 (3)	0.28079 (12)	0.0503 (5)	
H16	0.2002	0.3068	0.2696	0.060*	
C26	−0.12471 (17)	0.5925 (3)	0.09730 (13)	0.0588 (6)	
H26	−0.0836	0.6590	0.1214	0.071*	
C1	0.13761 (16)	0.3869 (3)	0.62522 (12)	0.0577 (6)	
C3	0.17622 (15)	0.4682 (3)	0.51528 (13)	0.0566 (6)	
H3	0.2138	0.5169	0.4929	0.068*	
C7	0.09049 (13)	0.4260 (3)	0.38996 (12)	0.0468 (5)	
H7	0.1237	0.4993	0.3747	0.056*	
C21	0.01022 (16)	−0.0003 (3)	0.16971 (14)	0.0549 (6)	
C18	0.25207 (16)	−0.0208 (3)	0.35107 (13)	0.0630 (7)	
H18	0.2942	−0.0649	0.3868	0.076*	
C6	0.06523 (15)	0.3289 (3)	0.58344 (12)	0.0562 (6)	
H6	0.0274	0.2823	0.6064	0.067*	
C23	0.01550 (14)	0.2770 (3)	0.06552 (12)	0.0587 (6)	
H23A	−0.0251	0.2010	0.0429	0.070*	
H23B	0.0330	0.3337	0.0267	0.070*	
C19	0.18501 (16)	−0.1081 (3)	0.31565 (13)	0.0611 (6)	
H19	0.1809	−0.2113	0.3273	0.073*	
C30	−0.16798 (15)	0.3466 (3)	0.04937 (13)	0.0584 (6)	
H30	−0.1566	0.2444	0.0410	0.070*	
C29	−0.24709 (15)	0.4006 (3)	0.02398 (14)	0.0625 (7)	
H29	−0.2884	0.3358	−0.0014	0.075*	
C27	−0.20378 (18)	0.6475 (3)	0.07271 (14)	0.0682 (7)	
H27	−0.2159	0.7495	0.0807	0.082*	
C2	0.19346 (16)	0.4570 (3)	0.59173 (14)	0.0636 (7)	
H2	0.2423	0.4964	0.6203	0.076*	
C13	−0.11773 (16)	0.0373 (3)	0.29201 (14)	0.0723 (8)	
H13A	−0.0963	−0.0271	0.3342	0.087*	
H13B	−0.1368	−0.0255	0.2488	0.087*	
H13C	−0.1627	0.0970	0.3012	0.087*	
C22	0.12756 (17)	0.0882 (3)	0.08263 (14)	0.0740 (8)	
H22A	0.0902	0.0055	0.0647	0.089*	
H22B	0.1737	0.0497	0.1191	0.089*	
H22C	0.1465	0.1322	0.0419	0.089*	
H1N	0.0456 (17)	−0.198 (3)	0.2158 (15)	0.082 (9)*	

### Atomic displacement parameters (Å^2^)

**Table:**

	*U*^11^	*U*^22^	*U*^33^	*U*^12^	*U*^13^	*U*^23^
Cl1	0.1218 (6)	0.0714 (4)	0.0461 (3)	−0.0034 (4)	−0.0010 (4)	−0.0014 (3)
Cl2	0.0795 (5)	0.1069 (6)	0.0911 (5)	0.0369 (5)	0.0232 (4)	0.0095 (5)
N1	0.0503 (10)	0.0402 (10)	0.0473 (10)	−0.0088 (8)	0.0085 (8)	0.0028 (8)
O1	0.0828 (12)	0.0474 (9)	0.0543 (10)	−0.0240 (9)	0.0167 (8)	−0.0025 (8)
C8	0.0432 (11)	0.0390 (11)	0.0447 (11)	0.0002 (10)	0.0124 (9)	−0.0023 (10)
F1	0.0623 (9)	0.0975 (12)	0.0743 (9)	−0.0097 (9)	−0.0015 (7)	0.0029 (9)
N3	0.0734 (15)	0.0359 (11)	0.0717 (14)	−0.0051 (11)	0.0170 (11)	−0.0048 (11)
C12	0.0506 (12)	0.0472 (13)	0.0442 (12)	−0.0026 (11)	0.0113 (10)	0.0036 (10)
C9	0.0455 (12)	0.0373 (12)	0.0486 (12)	−0.0024 (10)	0.0127 (10)	−0.0008 (10)
O2	0.0784 (12)	0.0592 (11)	0.0723 (11)	−0.0169 (10)	0.0025 (10)	−0.0181 (9)
N2	0.0589 (11)	0.0572 (12)	0.0434 (10)	−0.0002 (10)	0.0162 (9)	−0.0012 (9)
C20	0.0606 (15)	0.0426 (12)	0.0537 (13)	0.0031 (12)	0.0205 (12)	−0.0020 (11)
C15	0.0500 (12)	0.0434 (12)	0.0432 (11)	−0.0005 (11)	0.0154 (10)	−0.0017 (10)
C5	0.0487 (13)	0.0594 (15)	0.0467 (13)	0.0006 (11)	0.0077 (10)	−0.0037 (11)
C10	0.0478 (12)	0.0371 (11)	0.0413 (11)	−0.0046 (10)	0.0104 (9)	−0.0008 (9)
C11	0.0487 (12)	0.0422 (12)	0.0459 (12)	−0.0047 (10)	0.0086 (10)	0.0018 (10)
C4	0.0475 (12)	0.0403 (12)	0.0463 (12)	0.0032 (10)	0.0082 (10)	−0.0038 (10)
C17	0.0491 (14)	0.0706 (17)	0.0517 (13)	−0.0016 (13)	0.0125 (11)	−0.0044 (13)
C25	0.0652 (14)	0.0380 (12)	0.0396 (11)	−0.0039 (11)	0.0125 (11)	0.0057 (9)
C28	0.0681 (16)	0.0607 (16)	0.0515 (14)	0.0134 (14)	0.0193 (12)	0.0090 (12)
C24	0.0575 (13)	0.0437 (12)	0.0442 (12)	−0.0103 (11)	0.0101 (10)	0.0011 (10)
C14	0.0555 (13)	0.0399 (12)	0.0442 (12)	−0.0048 (10)	0.0124 (10)	−0.0037 (10)
C16	0.0539 (13)	0.0502 (13)	0.0493 (12)	−0.0017 (12)	0.0173 (11)	−0.0004 (11)
C26	0.0792 (17)	0.0440 (13)	0.0509 (13)	−0.0030 (13)	0.0103 (12)	−0.0024 (11)
C1	0.0786 (17)	0.0429 (13)	0.0457 (12)	0.0062 (13)	0.0019 (12)	−0.0018 (11)
C3	0.0594 (15)	0.0506 (13)	0.0575 (15)	−0.0081 (12)	0.0087 (12)	−0.0037 (12)
C7	0.0472 (12)	0.0440 (12)	0.0508 (13)	−0.0016 (10)	0.0142 (10)	−0.0016 (10)
C21	0.0652 (16)	0.0441 (14)	0.0570 (14)	−0.0068 (12)	0.0172 (13)	−0.0107 (12)
C18	0.0652 (16)	0.0706 (18)	0.0539 (14)	0.0174 (14)	0.0151 (12)	0.0086 (13)
C6	0.0616 (15)	0.0574 (15)	0.0500 (13)	0.0031 (12)	0.0137 (12)	0.0013 (12)
C23	0.0654 (15)	0.0668 (16)	0.0437 (12)	−0.0009 (13)	0.0123 (11)	0.0007 (12)
C19	0.0769 (17)	0.0476 (14)	0.0632 (15)	0.0096 (14)	0.0250 (13)	0.0059 (13)
C30	0.0679 (16)	0.0395 (13)	0.0615 (14)	−0.0006 (12)	0.0015 (12)	0.0005 (11)
C29	0.0621 (16)	0.0540 (15)	0.0657 (15)	−0.0020 (13)	0.0029 (13)	0.0061 (13)
C27	0.095 (2)	0.0495 (15)	0.0602 (15)	0.0153 (15)	0.0188 (15)	−0.0031 (13)
C2	0.0702 (16)	0.0525 (14)	0.0582 (15)	−0.0077 (13)	−0.0056 (13)	−0.0046 (12)
C13	0.0798 (18)	0.0659 (17)	0.0662 (16)	−0.0311 (15)	0.0068 (14)	0.0128 (14)
C22	0.0839 (19)	0.0835 (19)	0.0605 (15)	0.0118 (16)	0.0286 (14)	−0.0052 (14)

### Geometric parameters (Å, °)

**Table:**

Cl1—C1	1.739 (2)	C17—C16	1.375 (3)
Cl2—C28	1.746 (3)	C25—C26	1.386 (3)
N1—C12	1.450 (3)	C25—C30	1.388 (3)
N1—C11	1.454 (3)	C25—C24	1.510 (3)
N1—C13	1.456 (3)	C28—C29	1.366 (3)
O1—C9	1.218 (2)	C28—C27	1.367 (4)
C8—C7	1.345 (3)	C24—C23	1.517 (3)
C8—C9	1.494 (3)	C24—H24	0.9800
C8—C12	1.502 (3)	C14—C21	1.572 (3)
F1—C17	1.365 (3)	C16—H16	0.9300
N3—C21	1.358 (3)	C26—C27	1.379 (3)
N3—C20	1.397 (3)	C26—H26	0.9300
N3—H1N	0.84 (3)	C1—C6	1.372 (3)
C12—H12A	0.9700	C1—C2	1.374 (4)
C12—H12B	0.9700	C3—C2	1.379 (3)
C9—C10	1.542 (3)	C3—H3	0.9300
O2—C21	1.219 (3)	C7—H7	0.9300
N2—C23	1.457 (3)	C18—C19	1.387 (3)
N2—C22	1.461 (3)	C18—H18	0.9300
N2—C14	1.469 (3)	C6—H6	0.9300
C20—C19	1.371 (3)	C23—H23A	0.9700
C20—C15	1.393 (3)	C23—H23B	0.9700
C15—C16	1.380 (3)	C19—H19	0.9300
C15—C14	1.513 (3)	C30—C29	1.379 (3)
C5—C6	1.380 (3)	C30—H30	0.9300
C5—C4	1.387 (3)	C29—H29	0.9300
C5—H5	0.9300	C27—H27	0.9300
C10—C11	1.537 (3)	C2—H2	0.9300
C10—C24	1.568 (3)	C13—H13A	0.9600
C10—C14	1.587 (3)	C13—H13B	0.9600
C11—H11A	0.9700	C13—H13C	0.9600
C11—H11B	0.9700	C22—H22A	0.9600
C4—C3	1.396 (3)	C22—H22B	0.9600
C4—C7	1.461 (3)	C22—H22C	0.9600
C17—C18	1.364 (4)		
			
C12—N1—C11	111.05 (16)	C15—C14—C21	100.68 (17)
C12—N1—C13	110.34 (18)	N2—C14—C10	102.30 (16)
C11—N1—C13	112.83 (17)	C15—C14—C10	119.19 (17)
C7—C8—C9	116.53 (19)	C21—C14—C10	112.84 (17)
C7—C8—C12	124.05 (19)	C17—C16—C15	117.6 (2)
C9—C8—C12	119.41 (17)	C17—C16—H16	121.2
C21—N3—C20	112.2 (2)	C15—C16—H16	121.2
C21—N3—H1N	123.9 (19)	C27—C26—C25	121.8 (2)
C20—N3—H1N	123.5 (19)	C27—C26—H26	119.1
N1—C12—C8	113.68 (17)	C25—C26—H26	119.1
N1—C12—H12A	108.8	C6—C1—C2	120.7 (2)
C8—C12—H12A	108.8	C6—C1—Cl1	119.1 (2)
N1—C12—H12B	108.8	C2—C1—Cl1	120.16 (19)
C8—C12—H12B	108.8	C2—C3—C4	122.0 (2)
H12A—C12—H12B	107.7	C2—C3—H3	119.0
O1—C9—C8	120.96 (19)	C4—C3—H3	119.0
O1—C9—C10	121.42 (19)	C8—C7—C4	130.8 (2)
C8—C9—C10	117.59 (18)	C8—C7—H7	114.6
C23—N2—C22	114.55 (18)	C4—C7—H7	114.6
C23—N2—C14	106.82 (16)	O2—C21—N3	125.6 (2)
C22—N2—C14	116.15 (19)	O2—C21—C14	126.4 (2)
C19—C20—C15	122.4 (2)	N3—C21—C14	107.9 (2)
C19—C20—N3	128.1 (2)	C17—C18—C19	119.3 (2)
C15—C20—N3	109.5 (2)	C17—C18—H18	120.3
C16—C15—C20	119.3 (2)	C19—C18—H18	120.3
C16—C15—C14	130.7 (2)	C1—C6—C5	119.1 (2)
C20—C15—C14	109.71 (19)	C1—C6—H6	120.4
C6—C5—C4	122.3 (2)	C5—C6—H6	120.4
C6—C5—H5	118.8	N2—C23—C24	102.47 (17)
C4—C5—H5	118.8	N2—C23—H23A	111.3
C11—C10—C9	105.19 (16)	C24—C23—H23A	111.3
C11—C10—C24	114.90 (17)	N2—C23—H23B	111.3
C9—C10—C24	110.89 (17)	C24—C23—H23B	111.3
C11—C10—C14	110.74 (16)	H23A—C23—H23B	109.2
C9—C10—C14	111.11 (16)	C20—C19—C18	118.0 (2)
C24—C10—C14	104.14 (16)	C20—C19—H19	121.0
N1—C11—C10	107.35 (16)	C18—C19—H19	121.0
N1—C11—H11A	110.2	C29—C30—C25	121.8 (2)
C10—C11—H11A	110.2	C29—C30—H30	119.1
N1—C11—H11B	110.2	C25—C30—H30	119.1
C10—C11—H11B	110.2	C28—C29—C30	119.2 (2)
H11A—C11—H11B	108.5	C28—C29—H29	120.4
C5—C4—C3	116.5 (2)	C30—C29—H29	120.4
C5—C4—C7	125.05 (19)	C28—C27—C26	119.2 (2)
C3—C4—C7	118.4 (2)	C28—C27—H27	120.4
C18—C17—F1	118.6 (2)	C26—C27—H27	120.4
C18—C17—C16	123.5 (2)	C1—C2—C3	119.3 (2)
F1—C17—C16	118.0 (2)	C1—C2—H2	120.4
C26—C25—C30	117.0 (2)	C3—C2—H2	120.4
C26—C25—C24	120.2 (2)	N1—C13—H13A	109.5
C30—C25—C24	122.8 (2)	N1—C13—H13B	109.5
C29—C28—C27	121.0 (2)	H13A—C13—H13B	109.5
C29—C28—Cl2	118.6 (2)	N1—C13—H13C	109.5
C27—C28—Cl2	120.4 (2)	H13A—C13—H13C	109.5
C25—C24—C23	116.08 (18)	H13B—C13—H13C	109.5
C25—C24—C10	115.64 (17)	N2—C22—H22A	109.5
C23—C24—C10	104.34 (17)	N2—C22—H22B	109.5
C25—C24—H24	106.7	H22A—C22—H22B	109.5
C23—C24—H24	106.7	N2—C22—H22C	109.5
C10—C24—H24	106.7	H22A—C22—H22C	109.5
N2—C14—C15	110.68 (17)	H22B—C22—H22C	109.5
N2—C14—C21	111.43 (17)		
			
C11—N1—C12—C8	−46.9 (2)	C24—C10—C14—N2	15.56 (19)
C13—N1—C12—C8	−172.82 (19)	C11—C10—C14—C15	−98.0 (2)
C7—C8—C12—N1	−162.6 (2)	C9—C10—C14—C15	18.5 (2)
C9—C8—C12—N1	18.2 (3)	C24—C10—C14—C15	137.96 (18)
C7—C8—C9—O1	−17.5 (3)	C11—C10—C14—C21	19.7 (2)
C12—C8—C9—O1	161.9 (2)	C9—C10—C14—C21	136.27 (18)
C7—C8—C9—C10	164.77 (18)	C24—C10—C14—C21	−104.29 (19)
C12—C8—C9—C10	−15.9 (3)	C18—C17—C16—C15	0.3 (3)
C21—N3—C20—C19	−178.2 (2)	F1—C17—C16—C15	−179.82 (18)
C21—N3—C20—C15	−0.7 (3)	C20—C15—C16—C17	−1.4 (3)
C19—C20—C15—C16	2.2 (3)	C14—C15—C16—C17	−174.3 (2)
N3—C20—C15—C16	−175.45 (19)	C30—C25—C26—C27	1.3 (3)
C19—C20—C15—C14	176.5 (2)	C24—C25—C26—C27	−178.5 (2)
N3—C20—C15—C14	−1.1 (2)	C5—C4—C3—C2	1.5 (3)
O1—C9—C10—C11	−139.5 (2)	C7—C4—C3—C2	179.8 (2)
C8—C9—C10—C11	38.2 (2)	C9—C8—C7—C4	179.3 (2)
O1—C9—C10—C24	−14.7 (3)	C12—C8—C7—C4	0.0 (4)
C8—C9—C10—C24	163.01 (17)	C5—C4—C7—C8	−18.4 (4)
O1—C9—C10—C14	100.6 (2)	C3—C4—C7—C8	163.5 (2)
C8—C9—C10—C14	−81.7 (2)	C20—N3—C21—O2	177.5 (2)
C12—N1—C11—C10	74.2 (2)	C20—N3—C21—C14	2.2 (3)
C13—N1—C11—C10	−161.35 (19)	N2—C14—C21—O2	−60.5 (3)
C9—C10—C11—N1	−66.3 (2)	C15—C14—C21—O2	−177.9 (2)
C24—C10—C11—N1	171.46 (16)	C10—C14—C21—O2	54.0 (3)
C14—C10—C11—N1	53.8 (2)	N2—C14—C21—N3	114.7 (2)
C6—C5—C4—C3	−1.2 (3)	C15—C14—C21—N3	−2.7 (2)
C6—C5—C4—C7	−179.3 (2)	C10—C14—C21—N3	−130.82 (19)
C26—C25—C24—C23	−138.5 (2)	F1—C17—C18—C19	−179.7 (2)
C30—C25—C24—C23	41.7 (3)	C16—C17—C18—C19	0.2 (4)
C26—C25—C24—C10	98.8 (2)	C2—C1—C6—C5	0.5 (4)
C30—C25—C24—C10	−81.1 (3)	Cl1—C1—C6—C5	178.68 (18)
C11—C10—C24—C25	18.4 (3)	C4—C5—C6—C1	0.2 (4)
C9—C10—C24—C25	−100.7 (2)	C22—N2—C23—C24	176.68 (19)
C14—C10—C24—C25	139.74 (18)	C14—N2—C23—C24	46.6 (2)
C11—C10—C24—C23	−110.3 (2)	C25—C24—C23—N2	−162.38 (18)
C9—C10—C24—C23	130.57 (18)	C10—C24—C23—N2	−33.9 (2)
C14—C10—C24—C23	11.0 (2)	C15—C20—C19—C18	−1.7 (3)
C23—N2—C14—C15	−166.58 (18)	N3—C20—C19—C18	175.5 (2)
C22—N2—C14—C15	64.2 (2)	C17—C18—C19—C20	0.5 (3)
C23—N2—C14—C21	82.3 (2)	C26—C25—C30—C29	−0.8 (3)
C22—N2—C14—C21	−46.9 (3)	C24—C25—C30—C29	179.1 (2)
C23—N2—C14—C10	−38.6 (2)	C27—C28—C29—C30	0.9 (4)
C22—N2—C14—C10	−167.77 (18)	Cl2—C28—C29—C30	179.04 (18)
C16—C15—C14—N2	57.8 (3)	C25—C30—C29—C28	−0.3 (4)
C20—C15—C14—N2	−115.70 (19)	C29—C28—C27—C26	−0.4 (4)
C16—C15—C14—C21	175.7 (2)	Cl2—C28—C27—C26	−178.46 (18)
C20—C15—C14—C21	2.3 (2)	C25—C26—C27—C28	−0.8 (4)
C16—C15—C14—C10	−60.4 (3)	C6—C1—C2—C3	−0.2 (4)
C20—C15—C14—C10	126.16 (19)	Cl1—C1—C2—C3	−178.34 (19)
C11—C10—C14—N2	139.59 (16)	C4—C3—C2—C1	−0.9 (4)
C9—C10—C14—N2	−103.89 (18)		

### Hydrogen-bond geometry (Å, °)

**Table:**

*D*—H···*A*	*D*—H	H···*A*	*D*···*A*	*D*—H···*A*
C11—H11B···O2	0.97	2.37	2.968 (3)	119
C23—H23A···O2	0.97	2.58	3.162 (3)	119
C24—H24···O1	0.98	2.26	2.787 (3)	113
N3—H1N···O1^i^	0.84 (3)	2.50 (3)	3.288 (3)	157 (3)

Symmetry codes: (i) *x*, *y*−1, *z*.
